# Benchmarking Public Sector Food Environment Policies in Iran: A Cross‐Sectional Expert Assessment Study to Address Implementation Gaps

**DOI:** 10.1002/hsr2.71246

**Published:** 2025-09-17

**Authors:** Saeid Sadeghian‐Sharif, Nasrin Omidvar, Fatemeh Mohammdi‐Nasrabadi, Mina Babashahi, Mohammad Esmaeil Motlagh, Zahra Abdollahi, Simin Haghravan

**Affiliations:** ^1^ Faculty of Nutrition Sciences and Food Technology, National Nutrition and Food Technology Research Institute Shahid Beheshti University of Medical Sciences Tehran Iran; ^2^ School of Nutrition and Food Sciences Shiraz University of Medical Sciences Shiraz Iran; ^3^ Department of Pediatrics. School of Medicine Ahvaz Jundishapour University of Medical Sciences Ahvaz Iran; ^4^ Iran Ministry of Health and Medical Education Tehran Iran

**Keywords:** food environment, food‐epi, Iran, non‐communicable disease, obesity, policy

## Abstract

**Background and Aims:**

The escalating burden of obesity and non‐communicable diseases (NCDs) in Iran presents a critical public health challenge, underscoring the need for evidence‐based policies that foster healthier food environments. This study aimed to comprehensively evaluate national‐level food environment policies and supporting infrastructure in Iran using the Healthy Food Environment Policy Index (Food‐EPI), benchmarked against international best practices, and to identify priority policy actions to address key implementation gaps.

**Methods:**

The Food‐EPI tool was adapted and validated for the Iranian context through expert consultation, including content and face validity assessments. A systematic review of 89 government policy documents was conducted. Thirty‐nine experts from academia, government, and civil society assessed the extent of implementation of 47 Food‐EPI indicators across 13 domains, using a 5‐point scale relative to international benchmarks. Qualitative content analysis of expert feedback was used to extract and synthesize proposed actions to strengthen the current policy landscape.

**Results:**

No policy indicators achieved a “high” implementation rating. The majority of indicators were rated as “low” or “moderate” level of implementation, with the highest scores observed for food composition targets, front‐of‐pack labeling, and nutrient declarations. Key weaknesses were identified in food promotion, retail regulation, food trade, governance, and transparency. Infrastructure support performed better than policy implementation, particularly in areas such as obesity and NCD monitoring. Experts proposed 48 priority actions emphasizing strengthened regulatory enforcement, enhanced monitoring and intelligence systems, improved intersectoral coordination, sustainable funding mechanisms, and increased public awareness to create a healthier food environment.

**Conclusion:**

This first Food‐EPI assessment in Iran reveals substantial opportunities to improve both policy content and implementation to support healthier food environments. The results offer a strategic roadmap for Iranian policymakers to strengthen nutrition governance and reduce diet‐related health inequities. Aligning national efforts with global best practices can accelerate progress toward the Sustainable Development Goals and improved population health outcomes.

## 1 Introduction

The global rise in obesity, non‐communicable diseases (NCDs), and related disparities [1] has led policymakers to prioritize healthier food environments as a key strategy to prevent these conditions and improve public health. Food environments encompass physical, economic, political, and sociocultural factors influencing food access, marketing, information, and quality [2]. Governments play a critical role by regulating the private sector and building supportive infrastructure [2].

In Iran, NCD deaths increased from 50.1% in 1990 to 83.5% in 2019 [3], with leading risk factors including high blood pressure, elevated BMI, and high fasting plasma glucose [4]. Obesity rates have risen from 6% in 1990 to over 20% in 2021, with 63.1% of adults overweight or obese [5, 6]. Dietary patterns have shifted from traditional diets toward higher intake of refined carbohydrates, saturated fats, added sugars, and processed foods, contributing to a dual burden of malnutrition and obesity‐related NCDs [7, 8]. To address this, Iran has committed to the Sustainable Development Goals [9] and has developed National Document on the Prevention and Control of NCDs (NDPCNCD) and the National Document on Nutrition and Food Security (NDNFS) with a specific focus on providing healthier food environments by implementing policies such as food labeling, taxation of unhealthy foods, and food reformulation [10, 11].

This study aims to comprehensively assess current public sector policies in Iran for creating healthy food environments, benchmarked against international best practices, and to identify actions to address existing policy gaps.

## 2 Methods and Materials

**Figure 1:**
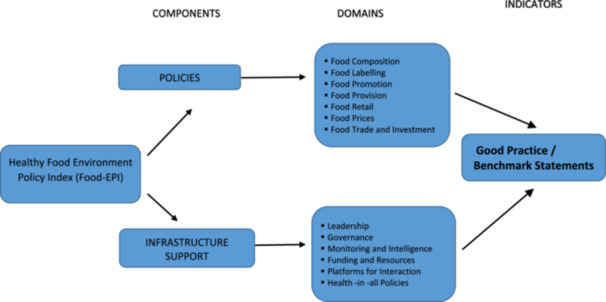
Components and domains of the Healthy Food Environment Policy Index (Food‐EPI).

We used the Healthy Food Environment Policy Index (Food‐EPI) framework developed by INFORMAS to benchmark Iran's food environment policies against global best practices [12]. The index consists of 47 indicators across 13 domains (Figure 1); details are in Supporting Information S1.

The Food‐EPI process in Iran involved six steps.

### 2.1 Validation of the Food‐EPI Tool

A forward‐backward translation was conducted and approved by the INFORMAS team. Content validity was assessed by 11 Iranian experts, calculating CVR and CVI scores using Lawshe's method (minimum acceptable CVR = 0.59; CVI threshold = 0.79), with some items revised based on feedback [13, 14]. Face validity was confirmed via expert ratings on a 5‐point Likert scale, retaining items with an impact score ≥ 1.5 [15].

### 2.2 Compilation of Evidence

National documents, policies, laws, and actions up to November 30, 2022, were collected from key institutions (e.g., Ministry of Health and Medical Education). Two experts independently reviewed and coded documents using deductive qualitative content analysis in MAXQDA 2020, following Food‐EPI domains and indicators. Information on regulations, implementation evidence, and best practices was summarized (Supporting Information S1).

### 2.3 Selection of Expert Panel

An 11‐member panel of specialists in nutrition, health policy, food science, and agriculture validated the tool. Additionally, 77 experts from academia (34), government (41), and NGOs (2) were invited to participate in the rating. Experts disclosed conflicts of interest before participation.

### 2.4 Validation of Evidence Report

Draft reports were circulated among panel members and government officials from the Supreme Council of Health and Food Security for feedback and verification.

### 2.5 Rating Process

Participants rated policy quality and implementation stage (agenda setting to evaluation) on a 5‐point scale indicating implementation percentage (0%–20% to 81%–100%). An option to select “unable to rate” was provided. Ratings reflected Iran's implementation relative to international benchmarks.

### 2.6 Identification of Proposed Actions

Panel members suggested policy modifications for each indicator. Two researchers analyzed these qualitatively, organizing them into proposed actions aligned with Iran's nutrition policies. The list was refined via meetings with stakeholders.

## 3 Data Analysis

Mean implementation scores were classified as “very limited” (0%–25%), “low” (25%–50%), “moderate” (50%–75%), or “high” (75%–100%). Indicators with over 20% “cannot rate” responses were excluded. Inter‐rater reliability was assessed using quadratic weighting in AgreeStat software (2013.1). Participation rate was 51%.

## 4 Ethical Approval and Informed Consent

The study was approved by the Ethics Committee of National Nutrition and Food Technology Research Institute (NNFTRI) (IR.SBMU. NNFTRI.REC.1400.051). Written informed consent was obtained from all participants after they were informed about the study objectives, procedures, and confidentiality measures.

## 5 Results

### 5.1 Validation of Food‐EPI Tool

All 11 experts participated in the validation process. The average CVR and CVI were 0.86 (range: 0.78–1) and 0.92 (range: 0.70–1), exceeding standard thresholds. Four indicators (provision3, retail1, retail4, fund3) required minor wording revisions based on expert feedback. No indicators were added or removed. All 47 indicators demonstrated satisfactory face validity with item impact scores > 1.5.

### 5.2 Evidence Included in the Evidence Report

Eighty‐nine policy documents were included as evidence of government actions. These comprised 5 constitutional policies, 20 parliamentary or high council laws, 5 national documents, 22 national guidelines or instructions, and 37 programs or acts. Documents were mapped to Food‐EPI domains: 42 to policy components, 35 to infrastructure, and 12 to both. A full list is available in Supporting Information S1.

## 6 Benchmarking Food Environment Policies Against International Best Practices

Thirty‐nine experts participated (17 from academic/NGOs, 22 from government). Inter‐rater reliability was high (ICC = 0.79; 95% CI: 0.71–0.89). Ratings did not differ significantly between independent and government‐affiliated experts.

**Figure 2:**
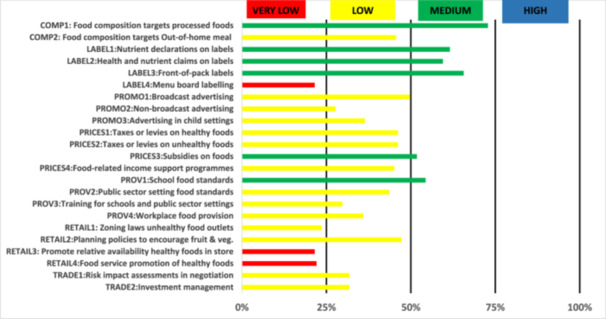
Level of implementation against the best international practices (Policies component).

In the “Policies” component (Figure 2), no indicator reached “high” implementation. Six were rated “medium,” 13 “low,” and 4 “very low”. The top indicators were “Food composition targets processed foods”, “Front‐of‐pack labeling”, and “Nutrient declarations on labels” with scores of 72.8%, 65.6%, and 61.6%, respectively. Conversely, the indicators with the lowest implementation levels were “Menu board labeling”, “Promote relative availability of healthy foods in store”, “Food service promotion of healthy foods”, and “Zoning laws for unhealthy food outlets” (all < 24%).

**Figure 3:**
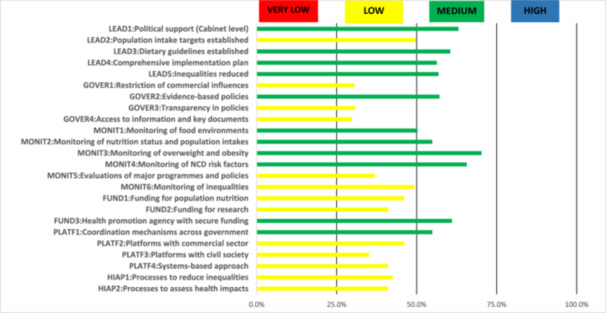
Level of implementation against the best international practices (Infrastructure component).

In the “Infrastructure” component (Figure 3), no indicator was rated “high” or “very low.” Eleven indicators were “medium”, and thirteen indicators were “low”. The highest ratings were for “Monitoring obesity,” “Monitoring NCD risk factors,” and “Political support” (70.3%, 65.6%, and 63.1%) and the lowest scores were in governance: “Access to government information,” “Restricting commercial influence,” and “Transparency” (29.7%, 30.6%, 30.8%, respectively).

## 7 Identifying Proposed Actions to Modify Policies

**Figure 4:**
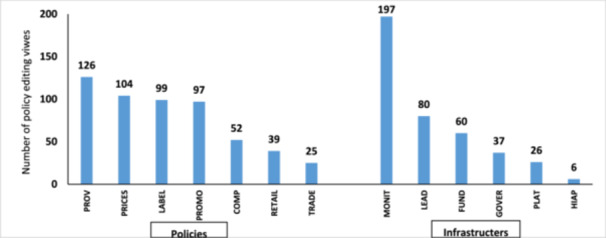
Distribution of policy editing views across policy and Infrastructure components, food composition domain; LABEL, food labelling domain; PROMO, food promotion domain; PRICE, food prices domain; PROV, food provision domain; RETAIL, food retail domain; TRADE, food trade and investment domain; LEAD, leadership domain; GOVER, governance domain; MONIT, monitoring and intelligence domain; FUND, funding and resources domain; PLAT, platforms for interaction domain; and HIAP, health in all policies domain. Details about each domain can be found in Supporting Information S1.

From expert input, 948 policy‐editing views were extracted—542 related to policies and 406 to infrastructure. Around 66% of these focused on five domains: “Monitoring,” “Provision,” “Labeling,” “Promotion,” and “Pricing” (Figure 4). After removing duplicates and consolidating related suggestions, these inputs were synthesized into 48 proposed actions (Table 1). The key recommendations spanned a broad range of strategic measures, including the establishment of independent monitoring systems for food promotion and taxation, revision of school food guidelines, and development of a national nutrition profiling model. Sustainable financing was also highlighted as critical for long‐term success. The panel emphasized increasing public awareness through media campaigns and improving food labeling standards. Additional recommendations targeted the restriction of unhealthy food advertising, enhancement of food reformulation policies, and expanded access to healthy foods in both retail and public settings. Strengthening political leadership and instituting regular performance reporting were also identified as crucial steps toward more effective policy implementation.

**Table 1:** The list of proposed actions to modify policies/infrastructure and numbers of their policy editing views, Food‐EPI IRAN. 2024.

Proposed actions to modify policies	Related domains	Number of policy editing views
Implement independent mechanisms to monitor policies related to food promotion and taxation of unhealthy foods.	PROMO, PRICES, MONIT	106
Review the Healthy School Canteen guideline to set regulations and monitoring for school and kindergarten food environments.	PROV, MONIT	74
Develop a Nutrition Profiling model to improve implementation of food environment policies like composition, promotion, school meals, and unhealthy food taxes.	COMP, PROMO, PROV, PRICES	56
Regularly report the population nutrition budget within the National Health Accounts.	FUND	46
Organize media campaigns to raise public awareness about food labeling.	LABEL	37
Update the Iranian Dietary Guidelines and Food Basket to support evidence‐based food policies.	LEAD	36
Expand support for healthy food in public and private centers and report pilot project outcomes.	PROV, MONIT	34
Design a standardized restaurant labeling system using simple symbols to show nutrition and allergens.	LABEL	33
Expand the school milk program to all primary schools.	PROV	32
Implement measures to strengthen field monitoring of the salt reduction program in traditional bread production.	COMP, MONIT	28
The president and cabinet should lead public health by resuming key NCD committee meetings and backing National Nutrition and Food Security initiatives.	LEAD	27
Conduct a study to simplify and standardize front‐of‐pack labeling for easy comparison of similar packaged foods.	LABEL	26
Establish a process to monitor and report on the ban of unhealthy outdoor food ads.	PROMO, MONIT	26
Increase micronutrient content and improve the nutritional and health characteristics of “National nutrition assistance programs”.	PRICES	23
Secure funding and set up independent monitoring for nutrition programs.	FUND	22
Evaluate the Electronic Food Coupon Plan.	PRICES, MONIT	22
Provide financial support to implement the Healthy School Canteen guideline in all primary schools.	PROV	21
Identify initiatives to include fresh fruits and vegetables in the “Electronic Food Coupon Plan”.	PRICES	19
Mandate chain supermarkets to improve healthy food access and limit unhealthy foods in stores.	RETAIL	19
Local markets and cooperative stores to boost healthy food access in food‐insecure areas.	RETAIL	18
Set voluntary or mandatory targets requiring chain restaurants to reduce fat, salt, sugar, and calories, and establish a system to monitor compliance.	COMP, MONIT	16
Establish laws requiring risk impact assessments before and during commercial agreement negotiations to minimize negative effects on population nutrition and health.	TRADE	16
Evaluation of “National Document on the Prevention and Control of NCDs”.	MONIT	16
Provide sustainable funding for regular, comprehensive food consumption surveys to monitor nutritional inequalities.	MONIT	16
Implement the National Food and Nutrition Surveillance Program to support evidence‐based policy‐making and monitor food environments.	MONIT	15
Enact laws banning all advertising of unhealthy foods and beverages targeted at children on websites, apps, and games.	PROMO	14
Require key stakeholders to report annual performance to SCHFS for public access.	GOVER	14
Reduce the prevention‐to‐treatment funding gap in the health budget.	FUND	14
Develop interactive platforms for government‐private sector coordination to promote a healthier food environment.	PLAT	13
Set clear population intake goals for key nutrients.	LEAD	12
Mandate stricter reformulation of children′s foods to reduce sugar, salt, trans fats, and boost beneficial nutrients.	COMP	9
Mandate broadcast media to promote healthy eating.	PROMO	9
Enforce plain packaging and ban cartoon characters on unhealthy foods.	PROMO	9
Engage municipalities to leverage their capacity for healthier food environments.	PLAT	9
Conduct a comprehensive study to identify policies that reduce prices of healthier foods like fruits, vegetables, and dairy.	PRICES	7
Increase the population nutrition budget in line with the burden of diet‐related NCDs.	FUND	7
Mandate formal health impact assessments in all food and nonfood policy proposals.	HIAP	6
Mandate nutrition expert‐approved meal plans in kindergartens.	PROV	5
Review the basic agricultural products list per the Iranian food basket and ensure commercial agreements protect strategic food stocks	TRADE	5
Require key stakeholders to use evidence‐based approaches in food and nutrition policy.	LEAD	5
Redefine serving‐size standards per dietary guidelines and promote voluntary compliance by food industries and chain restaurants, including offering half‐portions.	COMP	4
Revise standards to limit fat levels in imported meats.	TRADE	4
MoHME surveys like STEPS and CASPIAN should report results by ethnicity and immigrant status.	MONIT	4
Evaluate SCHFS and its branches to improve cross‐sector coordination for effective food and nutrition policies.	PLAT	4
Simplify nutrition claims rules for better understanding.	LABEL	3
Create opportunities to employ community nutritionists in public sector health promotion and disease prevention.	FUND	3
Conduct a feasibility study to design zoning laws restricting fast food density and location in communities.	RETAIL	2
Analyze government research funding for food environment improvement and NCD reduction to support its development	FUND	2

Abbreviations: COMP, food composition domain; FUND, funding and resources domain; GOVER, governance domain; HIAP, health in all policies domain; LABEL, food labelling domain; LEAD, leadership domain; MONIT, monitoring and intelligence domain; PLAT, platforms for interaction domain; PROMO, food promotion domain; PRICE, food prices domain; PROV, food provision domain; RETAIL, food retail domain; TRADE, food trade and investment domain. Details about each domain can be found in Supporting Information S1.

## 8 Discussion

Using the Food‐EPI tool, this study found that although Iran has developed a broad landscape for healthier food environment policies, none of the indicators were rated “high” for implementation; most fell into the low or very low categories. Infrastructure support appeared stronger than policies, with 46% of indicators rated at a medium level and none very low—suggesting a reasonable foundation for future efforts. Key initiatives include the SCHFS [17], national nutrition strategies (NDPCNCD and NDNFS) [9, 10], dietary guidelines [18], and national surveys [19]. As shown in Table 2, compared with other countries that implemented Food‐EPI between 2018 and 2024 [20, 21, 22, 23, 24, 25, 26, 27, 28, 29], including Ireland [24], Singapore [28], India [27], Norway [22], and Brazil [29], Iran′s infrastructure performance remains modest, primarily due to weaknesses in governance, funding, and monitoring, as well as leadership gaps (e.g., population intake targets, policy evaluations, and equity monitoring). Notably, Iran's strongest medium‐level indicators were political leadership and comprehensive NCD plans, reflecting top‐level commitment.

**Table 2:** Compare Iran's implementation of food environment infrastructure support with other countries that completed the Food‐EPI process between 2018 and 2024^c^.

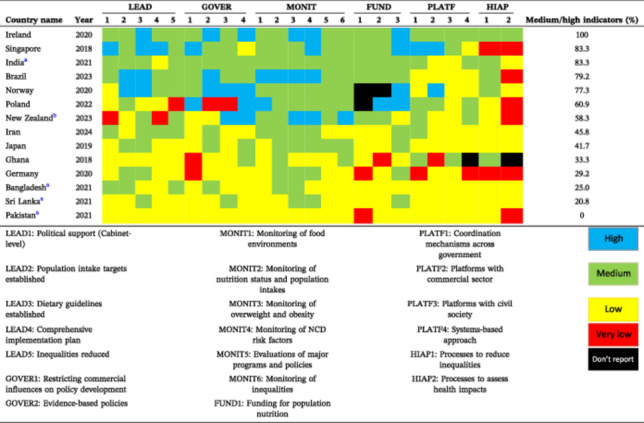																									

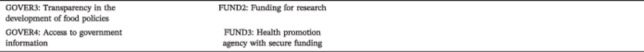			

^a^ The mean implementation level for benchmarked indicators is reported on a scale of 0–5, classified as: > 1.25 = very low, 1.25–2.5 = low, 2.5–3.75 = medium, 3.75–5 = high.

^b^ New Zealand′s Food‐EPI was evaluated and reported in both 2020 and 2023; only the most recent (2023) report is included in the table.

^c^ The 2020 Dutch Food‐EPI report is excluded due to differences in rating classification.

Roughly 75% of policy indicators were assessed as low or very low, particularly in food promotion, retail, trade, and provision. Despite legal efforts to restrict unhealthy food advertising and taxing unhealthy products [30, 31], enforcement challenges and lack of clear operational frameworks have limited their effectiveness [30, 31, 32, 33]. Ongoing barriers such as stakeholder resistance, unclear responsibilities, and weak oversight continue to hinder implementation [34]. Strengthening monitoring and enforcement is thus critical to improve accountability and ensure more effective policy execution.

Similar to other countries, policy domains such as food retail, trade, and menu labeling are challenging and consistently receive low implementation ratings, as reflected in Table 3. These areas tend to be challenging globally, reflecting the complexity of regulating food environments across diverse sectors. Iran's low scores in food pricing and provision—key to influencing behavior and access—highlight gaps when compared to high performers, for example, Norway [22], Poland [23], Brazil [29], and Ireland [24]. Recurring barriers to policy implementation identified in previous research [34]—including limited resources, weak governance, industry opposition, low awareness, and technical constraints—were echoed in expert recommendations.

**Table 3:** Compare Iran's implementation of food environment policies with other countries that completed the Food‐EPI process between 2018 and 2024^c^.

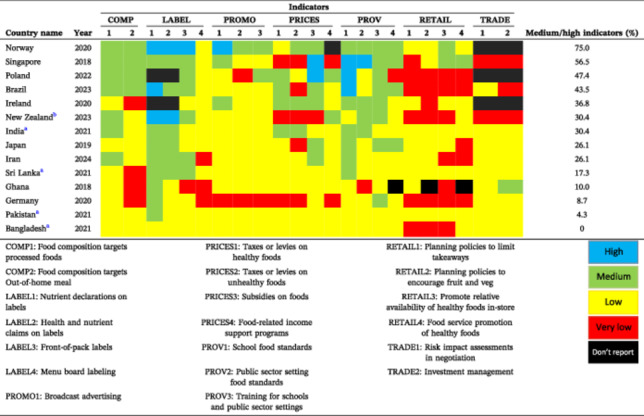																									

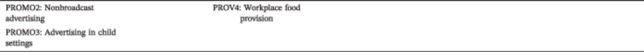			

^a^ The mean implementation level for benchmarked indicators is reported on a scale of 0 to 5, classified as: > 1.25 = very low, 1.25–2.5 = low, 2.5–3.75 = medium, 3.75–5 = high.

^b^ New Zealand's Food‐EPI was evaluated and reported in both 2020 and 2023; only the most recent (2023) report is included in the table.

^c^ The 2020 Dutch Food‐EPI report is excluded due to differences in rating classification.

Accordingly, 48 actionable recommendations were prioritized, focusing on regulatory enforcement, independent monitoring, intersectoral coordination, and sustainable funding. Raising public awareness through media and improving labeling standards were emphasized. Additional proposals included restricting unhealthy food marketing, improving food reformulation, and increasing healthy food access in retail and public settings. Stronger leadership and routine reporting were also deemed vital. Interestingly, proposed actions focused on visible and impactful domains like labeling, school food, promotion, monitoring, and pricing [16]—while areas such as retail, trade, governance, and cross‐sector platforms received less attention. This imbalance points to overlooked domains needing targeted engagement and resources to achieve a more comprehensive and WHO‐aligned policy framework [35].

This study provides evidence‐based guidance for stakeholders, paving the way for future implementation. The project's final phase—prioritizing proposed actions by experts and officials—offers a key opportunity to advance Iran's food and nutrition agenda and reduce diet‐related health inequities.

## 9 Conclusion

This study provides the first in‐depth evaluation of the implementation of food environment policies in Iran, utilizing the internationally recognized Food‐EPI framework. The results reveal that although the basic infrastructure supporting nutrition policy is fairly well developed, the execution of key policies is still inadequate, especially concerning food promotion, pricing, supply, retail, and trade sectors. Even with existing national plans and legal structures, the effectiveness is hindered by weak enforcement, fragmented governance systems, and a lack of proper monitoring. An expert panel put forward 48 practical recommendations, highlighting the importance of improving regulatory enforcement, creating independent monitoring bodies, ensuring stable funding sources, and increasing public involvement. If these suggestions are prioritized and implemented, they could guide the enhancement of nutrition policies in Iran. To achieve lasting improvements, emphasis should be placed on transforming policy promises into practical actions, encouraging cross‐sector collaboration, and overcoming systemic obstacles to healthier food environments.

## 10 Strengths and Limitations

The study's main strength lies in using the Food‐EPI tool, which engages experts and stakeholders to assess government efforts in promoting healthier food environments through a structured policy framework. While expert diversity is valuable, specialization in limited domains may introduce bias, though balanced representation was sought. Using international best practices, provides useful benchmarks but poses challenges due to limited evaluations. Additionally, the Food‐EPI framework's scope excludes issues like food smuggling—highlighted by experts as relevant in Iran [36]—due to the lack of established global benchmarks and indicators.

Another limitation is the variability in definitions of “healthy food” across contexts. For instance, some evidence links Maillard reaction products—formed during high‐heat cooking—to oxidative stress and inflammation [37]. While not the focus here, such perspectives broaden understanding of policy‐related challenges.

## Author Contributions

**Saeid Sadeghian‐Sharif:** conceptualization, data curation, formal analysis, funding acquisition, investigation, methodology, project administration, software, validation, visualization, writing – original draft, writing – review and editing. **Nasrin Omidvar:** conceptualization, data curation, formal analysis, funding acquisition, investigation, methodology, project administration, resources, supervision, validation, visualization, writing – original draft, writing – review and editing. **Fatemeh Mohammdi‐Nasrabadi:** data curation, formal analysis, funding acquisition, investigation, validation, visualization, writing – review and editing. **Mina Babashahi:** data curation, formal analysis, investigation, validation, visualization, writing – review and editing. **Mohammad Esmaeil Motlagh:** data curation, formal analysis, funding acquisition, investigation, methodology, resources, validation, visualization, writing – review and editing. **Zahra Abdollahi:** data curation, funding acquisition, investigation, resources, supervision, validation. **Simin Haghravan:** data curation, funding acquisition, methodology, resources, validation.

## Conflicts of Interest

The authors declare no conflicts of interest.

## Transparency Statement

The corresponding author, Nasrin Omidvar, affirms that this manuscript is an honest, accurate, and transparent account of the study being reported; that no important aspects of the study have been omitted; and that any discrepancies from the study as planned (and, if relevant, registered) have been explained.

## Supporting information

Supporting file 3.

## Data Availability

The authors confirm that the data supporting the findings of this study are available within the article and its supporting materials.
