# Supplementary material for: Benchmarking Public Sector Food Environment Policies in Iran: A Cross‐Sectional Expert Assessment Study to Address Implementation Gaps
Source: Health Sci Rep. 2025 Sep 17;8(9):e71246. doi: 10.1002/hsr2.71246 (PMC12441741; doi:10.1002/hsr2.71246)
Supplement: Supplementary file 1 — Supporting file 3. [file HSR2-8-e71246-s001.docx]

Benchmarking Public Sector Food Environment Policies in Iran: A Cross-Sectional Expert Assessment Study to Address Implementation Gaps (supplementay file)

**1. Definitions and scopes good practices indicators Iranian healthy Food Environment Policy Index**

| **COMP1** | **Difinition:**  Government has prepared food composition standards for different processed foods and determined the maximum amount of the nutrients of concern, especially if those foods are the main sources of the nutrients of concern (like trans fats and added sugars in processed foods, salt in breads, saturated fat in commercial frying oils).  **Scope:**   - Includes mandatory or voluntary targets, standards (i.e. reduce by X%, maximum mg/g per 100g or per serving) on nutrients of concern (include salt, simple sugar, SFA and TFA) for packaged industrial foods - Excludes legislated restrictions related to other ingredients (e.g. additives , pesticide residues) - Excludes mandatory food composition regulation related to other nutrients (e.g. Iron,folic acid or iodine fortification) - Excludes food composition of out of home meals (see COMP2) |
| --- | --- |
| **COMP2** | **Difinition:**  Government has prepared food composition standards for out-of-home meals at food service facilities and determined the maximum amount of the nutrients of concern, especially if those foods are the main sources of the nutrients of concern (trans fats, added sugars, salt, saturated fat)**.**  **Scope:**   - Includes mandatory or voluntary targets, standards (i.e. reduce by X%, maximum mg/g per 100g or per serving) on nutrients of concern (include salt, simple sugar, SFA and TFA) for out of home foods or meals ( e.g dine-in restaurants, bakery,dairy shops and etc) |
| **LABEL1** | **Difinition:**  On the labels of all packaged foods there are ingredient lists and nutrient declarations according to codex recommendations.  **Scope:**   - Includes mandatory or voluntary presentation of nutrient content list on all packaged industrial foods |
| **LABEL2** | **Difinition:**  There are strong and evidence-based regulatory systems for approving/studying food claims to protect consumers against misleading health and nutrition claims.  **Scope:**   - Includes mandatory or voluntary presentation of nutrition claims (e.g without or low calorie) and health claims (e.g regulation and strengthen of immune system) |
| **LABEL3** | **Difinition:**  On all packaged food, there are single, consistent, interpretive and evidence-informed nutrition information systems, so that consumer can easily assess the healthiness (nutritional quality) of food products.  **Scope:**   - Includes mandatory or voluntary presentation of nutrition information of packaged foods (e.g traffic light labelling for showed the amount of energy, salt, sugar, total fat and trans fatty acid per portion of food or per 100 grams/ml ) |
| **LABEL4** | **Difinition:**  For labeling menu-boards in fast-food chain restaurants, the government uses a single , consistent, simple and easily visible system to help consumers in interpreting energy and nutrients quality of on-sale foods and meals.  **Scope:**   - Includes present any nutrition information such as total kilocalories; percent daily intake; traffic light labelling; specific amounts of nutrients of concern or healthy choice symbol on the restaurant menu boards |
| **PROMO1** | **Difinition:**  The government implements effective policies to restrict exposure and power of unhealthy foods advertisements through broadcasting media (TV, radio).  **Scope:**   - Includes mandatory policy (i.e. legislation or regulations) or voluntary standards, or guidelines to restrict broadcasting of unhealthy foods advertisements through broadcast media (TV, radio) |
| **PROMO2** | **Difinition:**  The government implements effective policies to restrict exposure and power of unhealthy foods advertisements through non-broadcasting media (e.g. internet, social media, food packaging, sponsoring the events, outdoor advertisements including around the schools).  **Scope:**   - Includes mandatory policy (i.e. legislation or regulations) or voluntary standards, or guidelines to restrict broadcasting of unhealthy foods advertisements through non-broadcasting media include : print (e.g. children’s magazines), online (e.g. social media, branded education websites, online games, competitions and apps), outdoors and on/around public transport (e.g. signage, posters and billboards), cinema advertising, product placement and brand integration (e.g. in television shows and movies), direct marketing (e.g. fundraising in schools, provision of show bags, samples or flyers), product design and packaging (e.g. use of celebrities or cartoons, competitions and give-aways) - Where the promotion is specifically in a children’s setting, this should be captured in PROMO3 |
| **PROMO3** | **Difinition:**  The government implements effective policies to ensure that unhealthy foods are not commercially advertised around children^'^s places ( like kindergartens, schools, cultural and sport events).  **Scope:**   - Includes mandatory policy (i.e. legislation or regulations) or voluntary standards, or guidelines to restrict broadcasting of unhealthy foods advertisements through Children’s settings include: areas in and around schools or kindergartens, sport, recreation and play areas/ venues/ facilities and cultural/community events where children are commonly present |
| **PRICES1** | **Difinition:**  For encouraging healthy food choices, healthy food taxes and levies are minimized as much as possible (for example, minimizing or omitting sale taxes, excises, value-added taxes, and/or import duties of fruits and vegetables)  **Scope:**   - Includes minimizing or omitting of foods taxation or foods import duty |
| **PRICES2** | **Difinition:**  For discouraging unhealthy food choices (e.g. sugar-added beverages, food high in nutrients of concern), possible tax and levies are in place and increased their relating prices as at least 10 percent. These incomes are invested in community health promotion.  **Scope:**   - Includes differential application of sale taxes, value-added taxes or import duty on high calorie foods or foods that are high in nutrients of concern (include salt,,simple sugar, SFA and TFA) |
| **PRICES3** | **Difinition:**  The goal of food subsidies, including infrastructure funding support (e.g. research and development, supporting markets or transporting systems) are in favour of healthy instead of unhealthy foods.  **Scope:**   - Includes agricultural input subsidies, such as free or subsidised costs for fertiliser, seeds - Includes programs that ensure that farmers receive a certain price for their produce to encourage increased food production - Includes grants or funding support for food producers (i.e. farmers, food manufacturers) to encourage innovation via research and development - Includes population level food subsidies at the consumer end (e.g. subsidising staples ) - Excludes incentives for encourage establishment of new fruits and vegetables stores (e.g Municipal Fruit and Vegetable market and rural weekly market, see RETAIL2) - Excludes food subsidies related to food assistance programs (see PRICES4) |
| **PRICES4** | **Difinition:**  The government ensures that the program of supporting food-related incomes, are focused on healthy foods.  **Scope:**   - Includes programs where individuals can utilise government-administered subsidies, vouchers or discounts in retail settings for specific food purchasing. - Excludes food subsidies at the consumer end (e.g. subsidising staples at a population level –see PRICES3) |
| **PROV1** | **Difinition:**  The government ensure that there are clear and consistent policies (including nutrition standards) in the food services of schools and kindergartens (canteens, foods at events, advertisements, vending machines etc.) for providing and promoting healthy food choices.  **Scope:**  • Schools include government and non-government primary and secondary schools (7 to 12 years) and kindergartens include early childhood care services 0 to 6 years  • Includes policies and nutrition standards to provide and promote healthy food choices or to limit or restrict the provision or promotion of unhealthy food choices  • Includes policies that relate to school breakfast programs, where the program is partly or fully funded, managed or overseen by the government |
| **PROV2** | **Difinition:**  The government ensure that there are clear and consistent policies for food services (canteens, foods at events, advertisements, vending machines, public procurement standards, etc.) in the public sectors, in order to provision and promotion of healthy food choices.  **Scope:**   - Public sector settings include: - Public sector workplaces - Government-funded or managed services where the government is responsible for the provision of food, including public hospitals, nursing homes and prisons - Government-owned, funded or managed services where the general public purchase foods including health services, parks, sporting and leisure facilities, community eventsetc.   • Excludes school and early childhood settings (see PROV1)  • Includes policies and nutrition standards to provide and promote healthy food choices or to limit or restrict the provision or promotion of unhealthy food choices  • Includes modifying ingredients to make foods and drinks more healthy, or changing the menu to offer more healthy options |
| **PROV3** | **Difinition:**  The government ensure that there are appropriate supportive and educational systems to help schools and other public organizations and food service providers in meeting policies and healthy foods services guidelines.  **Scope:**   - Includes support for early childhood education services as defined in PROV1 - Public sector organisations includes settings defined in PROV2 - Support and training systems include guidelines, recipes and menu planning tools, expert advice, menu and product assessments and training workshops or courses |
| **PROV4** | **Difinition:**  The government actively encourage and support private companies to healthy foods and meals at their working places.  **Scope:**  • Includes policies and support and training systems to the provision of food in a workplace in ‘private companies’ includes profit companies |
| **RETAIL1** | **Difinition:**  Zoning laws and policies are robust enough to be used by local authorities to enforce restrictions on the density and location of fast food restaurant or other center of presenting unhealthy foods in communities as needed.  **Scope:**  • Includes government guideline that sets the policy objective of considering public health when reviewing and approving fast food planning applications |
| **RETAIL2** | **Difinition:**  Zoning laws and policies are robust enough to be used by local authorities to encourage establishment of new fruits and vegetables stores, as needed**.**  **Scope:**   - Outlets include supermarkets, produce markets, farmers’ markets, greengrocers, food cooperativess   Includes governmental/local policies to creating or developing these outlets or providing some facilities for encouraging private sector investment in this field |
| **RETAIL3** | **Difinition:**  The government ensure presence and access to supporting systems which encourage food stores to improve access to healthy foods and restrict to unhealthy foods**,** inside the stores**.**  **Scope:**   - Food stores include supermarkets, convenience stores, greengrocers and other speciality food retail outlets - Support systems include guidelines, resources or expert support - In-store promotion includes the use of key promotional sites such as end-of-aisle displays, checkouts and island bins as well as the use of shelf signage, floor decals or other promotional methods - In-store availability includes reducing or increasing supply (volume) of a product such as reducing the amount of shelf-space dedicated to sugar-sweetened drinks and confectionary, or offering fresh produce in a convenience store |
| **RETAIL4** | **Difinition:**  The government ensure presence of supporting systems which encourage food stores promotion and access to healthy foods and decrease promotion and availability of unhealthy foods  **Scope:**   - Food service outlets include restaurants, Fastfood restaurants or take-away restaurants - Support systems include guidelines, resources or expert support - Excludes settings owned or managed by the government (see PROV2 and PROV4) - Includes the strategic placement of foods and beverages in cabinets, fridges, on shelves or near the cashier - Includes the use of signage to highlight healthy options or endorsements (such as traffic lights or a recognised healthy symbol) |
| **TRADE1** | **Difinition:**  The government undertakes risk impact assessments before and during negotiations of commercial and investment agreements, tries to recognize, evaluate and minimize the direct and indirect negative effects of such agreements on population nutrition and health.  **Scope:**   - Includes policies or procedures that guide the undertaking of risk impact assessments before or during negotiation to assess risks and benefits in relation to public health and population nutrition - Includes policies or procedures that guide the evaluation of trade and investment agreements after an agreement is finalised to monitor the impact for the purpose of informing future negotiations or reviews - Includes policies or procedures that guide public consultation procedures before and during negotiations |
| **TRADE2** | **Difinition:**  The government uses its investment management position and supervising capacity to appreciate community nutrition and health in designing commercial and economic agreements.  **Scope:**   - Includes provisions in trade or economic agreements that protect the capacity of government to implement domestic policy in relation to food environments. This includes protections with respect to tariffs, non-tariff measures (such as quotas, regulations, standards, testing, certification, licensing procedures) and measures related to foreign direction investment |
| **LEADER1** | **Difinition:**  There is a strong and visible political support (at president/cabinet level) for improving food environments, community nutrition, diet- relate NCDs and their related inequalities.  **Scope:**   - Visible support includes statements of intent, election commitments, establishing priorities and targets, demonstration of support in the media, other actions that demonstrate support for new or strengthened policy documents that contain evidence of strong political support include media releases, speeches, pre-election policy papers, introduction of a bill, State-level strategic plans with targets or key performance indicators |
| **LEADER2** | **Difinition:**  For meeting recommendations the national and WHO recommended food intakes, the government has defined the clear population intake goals for the concerned nutrients.  **Scope:**   - Includes targets which specify population intakes according to average reductions in percentage or volume (e.g. mg/g) for salt, saturated fat, trans fats or added sugars - Typically requires the government to establish clear dietary guidelines on the maximum daily intake of nutrients of concern |
| **LEADER3** | **Difinition:**  Clear, interpretive and evidence-based food based dietary guidelines have been developed and implemented**.**  **Scope:**   - Food-based dietary guidelines should be for both genders and key age groups including infants and pregnant women - Evidence-informed includes extensive review of up-to-date research and mechanisms to seek expert input |
| **LEADER4** | **Difinition:**  There is comprehensive, clear and updated executive plan (including priority strategies, social marketing for public awareness and legal pressures for voluntary approaches ) linked to the national requirements and priorities for improving food environments and reducing the concerned nutrients, to reach to recommended food intakes and to reduce diet- relate NCDs.  **Scope:**   - Includes documented plans with specific actions and interventions (i.e. policies, programs, partnerships) - Plans should be current (i.e. maintain endorsement by the current government) - Plans should refer to actions to improve food environments (as defined in the policy domains above) and should include both policy and program strategies - Excludes overarching frameworks that provide general guidance and direction |
| **LEADER5** | **Difinition:**  The government's priorities for reducing inequalities or protecting the vulnerable groups in relation to diet, nutrition, obesity and NCDs have been defined.  **Scope:**   - Frameworks, strategies or implementation plans specify aims, objectives or targets to reduce inequalities including taking a preventive approach that addresses the social and environmental determinants of health - Frameworks, strategies or implementation plans identify vulnerable populations or priority groups - Implementation plans specify policies or programs that aim to reduce inequalities for specific population groups |
| **GOVER1** | **Difinition:**  There are strong procedures for restricting commercial Influences in developing food environments related policies, when they are in conflict of interests with community nutrition.  **Scope:**   - Includes government policies, guidelines, codes of conduct or other mechanisms to guide actions and decision-making by government employees, for example conflict of interest declaration procedures - Includes publicly available, up-to-date registers of lobbyist and/or their activities |
| **GOVER2** | **Difinition:**  For using evidences in developing food policies appropriate procedure are implemented.  **Scope:**   - Includes policies, procedures or guidelines to support government employees in the use of evidence for policy development including making vatity of structures in order to produce or ghater of evidences and providing facilities to accsibility, awareness and using of them - Includes policies, procedures or guidelines that stipulate the requirements for the establishment of a scientific or expert committee to inform policy development |
| **GOVER3** | **Difinition:**  For ensuring Transparency in developing food policies, appropriate procedures are implemented.  **Scope:**   - Includes policies or procedures to guide the online publishing of private sector and civil society submissions to government around the development of policy and subsequent government response to these - Includes policies or procedures that guide the use of consultation in the development of food policy - Include policies or procedures to guide public communications around all policies put forward but not progressed |
| **GOVER4** | **Difinition:**  The government ensures public access to comprehensive nutritional information and key documents (e.g. budget documents, annual performance reviews and health indicators)  **Scope:**   - Includes policies, procedures and structure to guide the timely, online publishing of government budgets, performance reviews, audits, evaluation reports or the findings of other reviews or inquiries - Includes ‘freedom of information’ legislation and related processes to enable the public access to government information on request, with minimal restrictions and exemptions - Includes policies or procedures to guide the timely, online publishing of population health data captured / owned by government |
| **MONIT1** | **Difinition:**  There are government's monitoring systems to regularly monitor food environments (especially for food compositions, regarding the concerned nutrients, food advertisements for children and nutritional quality of schools and other public sector's foods) against codes/protocols/standards/goals.  **Scope:**   - Includes monitoring systems funded fully or in part by government that are managed by an academic institution or other organisation - Includes regular monitoring and review of the impact of policies implemented by the government on food environments , in particular: - Monitoring of compliance with voluntary food composition standards related to nutrients of concern in packaged food products or out-of-home meals (as defined in the ‘Food composition’ domain) - Monitoring of compliance with food labelling regulations (as defined in the ‘Food labelling’ domain above) - Monitoring of unhealthy food promoted to children via broadcast and non-broadcast media and in children’s settings (as defined in the ‘Food promotion’ domain above) - Monitoring of compliance with food provision policies in schools, early childhood services and public sector settings (as defined in the ‘Food provision’ domain above) |
| **MONIT2** | **Difinition:**  There are regular monitoring of children and adults nutrition status and community's food intake, comparing to specific consumption targets or daily recommended amounts of consumption.  **Scope:**   - Includes monitoring of adult and child intake in line with the Iranian Dietary Guidelines - Includes monitoring of adult and child intake of nutrients of concern - ‘Regular’ is considered to be every five years or more frequently |
| **MONIT3** | **Difinition:**  There is regular monitoring of the prevalence of overweight and obesity among children and adults, using anthropometric measurements.  **Scope:**   - Includes monitoring of other NCD risk factors (not already covered by ‘MONIT1’, ‘MONIT2’ and ‘MONIT3’) include level of physical activity, smoking, alcohol consumption and diet-related NCDs include hypertension, hypercholesterolaemia, Type 2 Diabetes, cardiovascular disease and diet-related cancers - ‘Regular’ is considered to be every five years or more frequently - May be collected through a variety of mechanisms such as population surveys or a notifiable diseases surveillance system |
| **MONIT4** | **Difinition:**  There is regular monitoring of the prevalence of the risk factors of NCDs and occurrence rates (e.g. prevalence, incidence, mortality) for the main diet- relate NCDs.  **Scope:**   - Includes a comprehensive evaluation framework and plan that aligns with the key preventive health or nutrition implementation plan |
| **MONIT5** | **Difinition:**  There is enough evaluation of the main policies and programs for measuring their efficacy and share in reaching to nutritional goals and health programs.  **Scope:**   - 'Population nutrition' includes promotion of healthy eating, and policies and programs that support healthy food environments for the prevention of obesity and diet-related NCDs - The definition excludes all one-on-one and group-based promotion (primary care, antenatal services, maternal and child nursing services etc.), food safety, micronutrient deficiencies (e.g. folic acid fortification) - The 'Population Nutrition' budget should include workforce costs (salaries and associated on-costs) and program budgets for the 2020-21 financial year (regardless of revenue source), reported separately. - The workforce comprises anyone whose primary role relates to population nutrition and who is employed full time, part time or casually by the Department of Health |
| **FUND1** | **Difinition:**  The budget of "improving Population nutrition", as a part of total budget of health affairs and/or related to the load of diet- relate NCDs, is adequate for reducing diet- relate NCDs.  **Scope:**   - Includes the clear identification of research budget related to improving food environments, reducing obesity, NCDs and their related inequalities in health or medical research strategies or frameworks for the 2020-21 financial year and comparing its changes during atleast 5 years |
| **FUND2** | **Difinition:**  The governmental research budget has dedicated to improvement of food environments, reduction of obesity, NSDs and their related inequalities.  **Scope:**   - Includes the clear identification of research priorities related to improving food environments, reducing obesity, NCDs and their related inequalities in health or medical research strategies orframeworks - Includes identifying research projects conducted or commissioned by the government specifically targeting food environments, prevention of obesity or NCDs (excluding secondary or tertiary prevention) - It is limited to research projects committed to or conducted within the last 12 months. - Excludes evaluation of interventions (this is explored in ‘MONIT5’ and should be part of an overall program budget) |
| **FUND3** | **Difinition:**  There is a legal health agency with assured financial process for improving community (Population ) nutrition  **Scope:**   - Agency was established through legislation - Includes objective to improve population nutrition in relevant legislation, strategic plans or on agency website - Secure funding stream involves the use of public budget or other secure source |
| **PLATF1** | **Difinition:**  There are strong coordination mechanisms in departments and different levels of government (local and national) to ensure the consistency and integrity of the policies of preventing obesity-related NCDs, food and diets, all over the government.  **Scope:**   - Includes strategic plans or frameworks that map the integration and alignment of multiple policies or programs across governments and across departments |
| **PLATF2** | **Difinition:**  There are official platforms between government and commercial food sector for implementing healthy food policies.  **Scope:**   - The commercial food sector includes food production, food technology, manufacturing and processing, marketing, distribution, retail and food service, etc. - Includes platforms to support, manage or monitor private sector pledges, commitments or agreements to implement policy   Excludes initiatives covered by RETAIL3 and RETAIL4. |
| **PLATF3** | **Difinition:**  There are official platforms for regular interactions between government and civil society and other strategies for improving community nutrition**.**  **Scope:**   - Civil society includes community groups and consumer representatives, NGOs, academia,professional associations, etc.   • Includes platforms for consultation on proposed plans, policy or public inquiries  • Excludes policies or procedures that guide consultation in the development of food policy (see GOVER3) |
| **PLATF4** | **Difinition:**  The government, along with local organizations, directs a broad, consistent, effective, integrated and sustainable approach for improving the health of food environment in national level.  **Scope:** |
| **HIAP1** | **Difinition:**  There are processes for ensuring that in designing all government's policies on food and community nutrition, health impacts and reducing health inequalities among vulnerable groups have been considered are in priority.  **Scope:**   - Includes policies, procedures, guidelines, tools and other resources that guide the consideration and assessment of nutrition, health outcomes and reducing health inequalities or health impacts in vulnerable populations prior to, during and following implementation of food-related policies - Includes the establishment of cross-department governance and coordination structures while developing food-related policies |
| **HIAP2** | **Difinition:**  There is regular monitoring of the prevalence of overweight and obesity among children and adults, using anthropometric measurements.  **Scope:**   - Includes a government-wide HiAP strategy or plan with clear actions for non-health sectors |

2. The list of relevant Documents/Acts taken by the Iranian Government for creating healthier food environments to reduce obesity, NCDs and their consequent inequalities

| **Constitutional Law/General Policies** | **National documents** | **National programs/Other Acts** |
| --- | --- | --- |
| 1. General health policies 2. General policies of the system 3. Constitution of Iran, 4. General policies of resistance economy 5. Iran's "Twenty-Year Vision Document | 1. National Documents for Prevention and Control of Non-Communicable Diseases (NDPCNCD) 2. National Documents on Nutrition and Food Security (NDNFS) 3. Fundamental reform document on education 4. National policy document on integrated early child development 5. Poverty reduction and targeting subsidies document | 1. Reduction of saturated and trans fatty acids in edible oils 2. National program to reduce the nutritional risk factors of industrials and commercials foods 3. Electronic Food Coupon Plan 4. Integrated system of foreign exchange transactions (NIMA system) 5. National Program for Improving Nutritional Status of Children 6. Nutritional assistance program for pregnant women 7. Feeding Program (Providing a Warm Meal in Rural Kindergartens) 8. Weight and Obesity Control of Students (Kouch) 9. The IRAN-Ending Childhood Obesity (IRAN-ECHO) Program 10. School Milk Program 11. Rural cooperative stores 12. Municipal fruit and vegetable markets 13. Local weekly markets 14. Iran's Health Transformation Plan 15. Prevention and control of NCDs and their risk factors in the Iran's HTP 16. Community nutrition improvement program in the Iran's HTP 17. Food security improvement program in highly food insecure provinces (BAGH project) 18. Subsidy program (cash transfer) 19. Indicators of Equity in Health 20. The National Food and Nutrition Surveillance Program 21. Establishment of National Institute of Health Researches (NIHR) 22. Health Information Observatory Dashboard 23. National Health Accounts (NHA) reports 24. National website to Dissemination of and Free Access to Information 25. Consumer Price Index 26. Awarding health-friendly logo 27. establishment of the "General Directorate of Non-Governmental Organizations (NGOs) and Health Charities" in the MoHME 28. The population-based nationwide STEPS surveys 29. The Comprehensive Survey on Iranian Food Consumption Patterns and Nutritional Status 30. Iranian Households Income and Expenditure surveys 31. Childhood and Adolescence Surveillance and Prevention of Adult Non-Communicable Disease in Iran (The CASPIAN surveys) 32. Urban HEART Studies 33. The third 5-year plan for the Tehran’s urban Development) 34. The Healthy Cities Project 35. Tehran municipal health campuses 36. National plan to monitoring non-communicable diseases nutritional risk factors in industrial and commercial foods 37. Health Impact Assessment (HIA) or Health Annex |
| **parliamentary law/Supreme Councils Approvals** | **National Regulations/ guidelines/ Instruction** |  |
| 1. The Permanent Sentences of the Country Development Plans law 2. The law of adding some articles to the financial regulations of the government 3. Direct taxes law 4. Value Added Tax Law 5. Iran’s six development plan 6. Subsidies targeting law 7. The law on objectives and duties of the Ministry of Education 8. Labor Law 9. The fourteenth meeting of the SCHFS 10. The Executive Regulations of Article 13 of the Food and Beverage Law 11. Trade Union Law 12. Foreign investment encouragement and support law 13. The low on formation MoHME (The Integration of Medical and Health Education and Health Care Services) 14. The law on establishing National Nutrition and Food Technology Research Institute (NNFTRI) 15. The Law on Dissemination of and Free Access to Information 16. Iranian National Budget law 2023-24 17. The law to formation of Iran Chamber of Commerce, Industries, Mines and Agriculture (ICCIMA) 18. The law on the organization, duties and election of the Urban and Rural Islamic Councils and mayors 19. Iran's comprehensive scientific map in the field of health | 1. Minimum Rules for Labeling Food and Beverage Products Guideline 2. Food labeling regulations with the words "low trans" or "trans free" 3. Policies and Regulations Governing on Environmental Advertising 4. The Set of Production Criteria for Television and Radio Advertising 5. Increase in palm oil import tariff 6. Social Welfare Umbrella Regulation 7. Regulations for providing, maintaining and promoting the physical, mental and social health of students 8. The regulations for the establishment and management of kindergartens 9. Health regulations of kindergartens 10. Healthy School Canteen (HSC) guideline 11. The instruction of Healthy cooking in corporate catering 12. The instruction of improving meal composition in the public sector corporate catering 13. Basic health services package for government employees 14. The standard of nursing homes 15. The Regulations of the hospital nutrition department 16. The Procedure for Issuing licenses for restaurants and other food outlets 17. The status of the Central Organization of Rural Cooperatives, duties 18. Iranian food-based dietary guideline (FBDG) 19. Iranian Society's Desirable Food Basket 20. Regulations of the Provincial Planning and Development Council 21. Health-Promoting School (HPS) Regulations 22. Executive instructions for monitoring food and beverage products |  |
